# Health, Wealth, and Religion

**Published:** 1854-12

**Authors:** 


### HALL'S JOURNAL OF HEALTH.

VOL. I.] DECEMBER, 1854. [NO. XII.

### HEALTH, WEALTH, AND RELIGION

Are the three grand duties of life. Each additional year confirms me in the opinion that pulpit teachings in reference to money are erroneous, mischievous, and inconsistent. The vanity of riches, that silver and gold are dross, that wealth is a snare, that it is hard for a rich man to enter heaven, these are stereotype themes and afford scope for a beautiful display of words and imagination.

If money is indeed so trashy, if its pursuit perils a man's soul, why is it that in some cases we never go to church on the Sabbath-day without having a silver plate handed round, that tells plainly enough whether the giver throws in a copper cent or a silver dollar, thus shaming the humble poor and tempting the ostentatious to go beyond their means; we thus give the lie to our teachings, and more than this, we practically ignore the expressive teachings of scripture, that we must not let the left hand know what our right hand doeth. I will leave it to the reflective man of wealth, who is yet among the world's people, if he does not often turn away with a feeling of contemptuousness at theory and practice so mal apropos.

At one moment we are told that wealth is a canker; how unavailing to procure happiness; the next we are reminded of how blessed a thing it is to give, and what a large good may be done in the judicious use of a small amount of money. These inconsistencies perplex the "feeble folk" and confuse the lambs of the flock, for whom we ought specially to care. Men of New York, and Philadelphia, and Boston, and of the thousands of smaller cities and towns of this broad land, whether money be a hindrance against entering heaven or not, judge ye; but this I know, its possession here is necessary to a seat near God's altar. We cannot sit under the droppings of the sanctuary if we are not rich, at least comparatively. It is notorious that the radical, the distinctive principles of primitive christianity are reversed among us. In early times, when the love of a recently ascended Saviour burned within the hearts of his followers, it was heralded abroad as something singular and almost miraculous, that

"*The poor have the gospel preached to them.*"

Such is not the case in New York. Here we must give a thousand dollars for a pew holding five persons, and in addition to that thousand we must pay seven per cent. every year for church expenses. In our Fifth Avenue Church, built at the cost of a hundred and thirty thousand dollars, there is not a pew or even single sitting to be had; this is largely complimentary to our minister, one of the best of men and of commanding talents, yet this is the very kind of man needed for the poor, for it requires all his piety to stoop so low as to wash their feet, and all his talents to make the hidden things of the Bible plain to their uncultivated minds; but the rich bid the highest, and the poor must put up with any crumbs they may get from the tables of their richer brethren in the way of standing room in the vestibule or gallery, or an occasional vacant seat on rainy days, or very dusty, or very warm, or very windy, or very cold, or in the dog days when it isn't fashionable to be seen in town; in this last case, indeed, there is plenty of room, but the voice of that pious and talented man is not there to instruct by thoughts that breathe and words that burn; still there is a voice there giving sound, coming from some weak brother, or practising licentiate, or high-falutin sophomore. I really don't know but after all our Quaker Friends are nearest primitive practices in this most important respect. Their houses of religious meeting are very large, very plain, very clean, and abundantly free to all who come; the "weary and the heavy laden" may indeed there literally find rest—rest for the weary body, rest in the decent quiet of the place, from the tumultuous tossings of the world's conflict with want, its strivings for bread. Next to them are the lovely *Moravian Brethren*, and then our Methodist friends, but they, alas, are receding before so-called *civilization*, or falling in with the fashion of the world, in selling places near God's altar to the highest bidder. Last month I went to a church iu Arch-street,

Philadelphia; the heads of the *three* aisles were crowded with people waiting for the *one sexton* to show them places; and we observed in several instances that when the owners came they took keys from their pockets and unlocked the pew doors. No doubt reasons are at hand for the fine church and for the pew system, but whether they will stand the test of universal brotherhood, all being children of the same common Father of all, who is himself no respecter of persons, I cannot say. The question comes back with some power, ought I to lock my pew door on a waiting brother? ought I to exclude the poor from the kingdom of heaven, by virtually excluding them from receiving those teachings which guide and prepare for that kingdom? These things may at least be *re-investigated.*

The general impression in a christian community is, that the first duty of man is to become a christian. A physician is naturally possessed of the confidence of his patients as to health, and gradually that is extended to other things as dear. I am often consulted as to marrying, and professional duty sometimes leads me to take the initiative as to advice on that subject; the first item of all is, "*Let the man or woman you marry be healthy.*" If your companion for life is ignorant, you may instruct; if poor, you may enrich; if wanting position, you may elevate; if lacking religion, you may place at hand the means of conversion—your own pious life will almost certainly be that means of conversion; if lazy, or dirty, or unmethodical, which is just as bad perhaps, you can correct these by example and by judicious and encouraging teachings; but if you marry a bad constitution, a radically diseased body, there is no effective certain remedy, and you lay the foundation for a life of disquietude, discouragement, and expense to yourself; while if any children are born to you, they will inherit that misfortune in an aggravated form, and thus you will have a sickly child to be a canker, a festering wound, a rankling thorn, a weary wasting anxiety, to the latest hour of life; for can anything throw one ray of sunlight across my heart when the child of my bosom is wasting and waning before me to a certain and premature grave? I think not.

To be truly religious, and to have true views on all subjects connected with religion, a man should have undisputed health. A sound mind in a sound body is, I believe, an axiom, a first principle, and as no child is born religious, and all "go astray from the womb, speaking lies," a healthfully acting mind in a healthy body seems to be a prerequisite towards giving the arguments for religious truth the consideration due them; if this be so, then the *first* parental *duty* to the new-born child is, not in reference to religion directly, but in reference to its health, its preservation if good, and its improvement if defective. It seems then to follow, that good health, other things being equal, is a prerequisite in the investigation of religious truth, and rather increases the probability that the arguments substantiating such truth will be properly appreciated; in other words, a healthy man is more likely to be brought under the power of religious truth, and to become a christian from sterling principle, than an unhealthy man. I know it is said by the blessed One himself, "Seek first the kingdom of God and his righteousness," but a search pre-supposes eyes to see, health to perceive the full force of exhortation, argument, miracle.

I will not take it upon me to run this out, therefore I go no further than to say, that the preservation and the promotion of the highest health of body is among the very first duties of an immortal mind; for, the better we can understand our duty here, the higher and the more glorious will be our position hereafter; and to be able to comprehend our duty in its broadest sense, and with the most convincing power, we ought to be able to bring to bear on that truth the greatest strength of mind which the highest health of body can secure.

If these things be so, where stands the man who pays no attention to the *preservation of his health?* Where stands the parent who gives no instruction to his children, nor causes them to be instructed, as to the laws of health and life? Does a good citizen even, let alone a christian parent, do his duty by his children, when he goes no further than the catechism, the confession, or the prayer book?

If, then, I were required to comprise in three parts the main effort of life, I would say—

*Strive with all the energy God hath given you, to be healthy, to be religious, to be rich.* The more healthy you are, the more truly and highly religious you can be; and the richer you are, the more can you do towards placing the same glorious religion within reach at least, of the perishing myriads of earth's poor. In view of this mode of argument, there is more truth than appears at a first glance, in the two following extracts from some of our exchanges, showing sarcastically how necessary wealth is to a wide influence, how it gives influence in spite of a bad private character, but how resistless and how wide, if piety and wealth went hand in hand for humanity and heaven.

What I have said is not so much against fine churches, as against the very general habit of bearing hard on riches and rich men in theory, while in practice all that is possible is done to cause the rich to bestow their riches on the church and its professed objects.

Another of the things to be *re-investigated*, as hinted in the November number, is simply this:—

Is it right for the pulpit to bear so hard from Sabbath to Sabbath on riches, and rich men, per se? More pains ought to be expended in a just discrimination between abused wealth, unsanctified wealth, and wealth itself. We say money is the sinew of war; not less true is it that money is the sinew of the war spiritual. The great Head of the Church has chosen to ordain that religion should be extended over the world not by miracle, but by money, and the instrument should be honored at least for the uses it is put to, and not fulminated against, as it is, almost with spitefulness sometimes as unwise as the railings of the rabble in the times of barricades, against the rich, and the titled, and the elevated.

Our newspapers, too, make it a standing and favorite theme to bespatter the rich, to anathemize them. Indeed, the unguarded are almost persuaded sometimes to consider it a crime to be rich. This is all wrong, radically wrong. Instead of cherishing a kind of malignant feeling against such men, we ought rather to go on the principle that to be rich, pre-supposes its possessor to have been a man of industry, of self-denial and of economy, therefore, in all justice, the very fact of a man being rich entitles him to our respect, for I am persuaded that more men are rich from inheritance and from economy and industry, than from dishonorable practices; and surely there is nothing discreditable in being wealthy either by inheritance or by industry. Away then with this railing against the rich, let it be preached from the pulpit, and let it be proclaimed by the press with its million tongues, that to accumulate wealth is one of the first, one of the highest, one of the noblest duties of an immortal mind, and then, that to use it benevolently makes that mind akin to God.

The true Christian doctrine is, *make all you can honorably, save all you can unmeanly, bestow all you can unostentatiously.*

There is truth then in "Lowell Philosophy," while in the article following it, there is that blending of the true, the specious and the false, which makes it almost *dangerous to admit the majority of weekly newspapers to our centre tables.* And although mine is a *Journal of Health*, yet I did not design originally that it should be restricted to bodily health, but contemplated an occasional episode for the promotion of health moral, social, political. And as a parent and citizen and church member, I throw out this incidental subject, another item for *re-consideration.* Look to your weekly newspaper and reflect whether it is always on the side of the Bible and Bible religion, or whether it does not more than semi-occasionally, aim side thrusts against that religion as now generally understood.

### Philosophy at Lowell.

Every man owes it to society to become rich, for the poor man's advice is never heeded, let it be ever so valuable. The more wise one may be, the more he owes it to his country to become wealthy. Every addition made to a man's fortune adds ten per cent to his influence. Let a man throw a doubloon on the counter, and every one will want to hear it ring. Throw a cent down, however, and its voice would prove no more attractive than a poor relation's.

### If you want your Talents Appreciated, get Rich.

"That tells the whole story in a nutshell. If you wish to be anybody in the estimation of mankind, get rich. No matter how pure your morality, how lofty your aspiration, how disciplined your mind, unless you have a fortune you will never be loved, noticed or respected. But if your ancestor chanced to be a miser, and thus left you a goodly heritage, you are fawned on, courted and flattered. If you are a real knave, or a blockhead, it's of no consequence, for you are rich. This blind idolatry of wealth, the worship of mammon, is enough to make an honest man blush for his race. The almighty dollar is the whole end of existence, and the only object of life. The minister of God forgets his high calling, and preaches for a higher salary. His congregation following him to the costly and magnificient edifice ostensibly dedicated to God, and instead of meditating on the true end of life, they are absorbed in admiring their own or envying their neighbor's rich garments, and scheming how the morrow shall add to their store of wealth.

Extravagance, fashion, and cheating, throng our streets, and jostle against honest toil. Liveried footmen and costly coaches hurry by and splash merit with mud thrown from the wheels —and thus it is in every phase of life. The toiling, laboring poor are despised and contemned. Riches are coveted, sought for and worshiped by the million. Honesty and truth, merit and talent, are sold for a 'mess of pottage.' Too often the most open dishonesty is forgiven and forgotten, because wealth blinds the eyes and obliterates the memory of the public. 'An honest man is the noblest work of God,' was once true; but now, 'get all you can, and keep what you get,' is the great principle of the age."

---

### RECIPE FOR A RAINY DAY.

We did not intend in the conduct of our Journal to take the bread out of our own mouths by telling people how they were to get well without calling on a doctor. Still, we made no promise to that effect; we merely stated our intention, thus leaving a hole for honorable exit in case of a *change of views*, for wise men do change sometimes, fools—never. We will take care to be always consistent and always safe by being lavish in the expression of our intentions, but penuriously parsimonious in proffering promises. As a doctor, we never promised to cure certainly man, woman or child of anything, not even of the scratch of a pin, nor do we ever expect to do so silly a thing. None but the verdant or the wicked ever make promises of cure; but to the recipe "*How to lay up money for a rainy day*," as obtained from a Kennebec paper, it being kept in mind that it is healthful and agreeable to have money over and above what you owe and need.

A number of years ago, Charles and Clara S—— were mar-

